# Carbohydrate-Based Fat Mimetics Can Affect the Levels of 3-Monochloropropane-1,2-Diol Esters and Glycidyl Esters in Shortbread Biscuits

**DOI:** 10.1007/s11130-019-00723-z

**Published:** 2019-03-05

**Authors:** Anna Sadowska-Rociek, Ewa Cieślik

**Affiliations:** Malopolska Centre of Food Monitoring, Faculty of Food Technology, University of Agriculture in Krakow, Balicka Street 122, 30-149 Krakow, Poland

**Keywords:** 3-monochloropropane-1,2-diol esters, 3-MCPDE, Glycidyl esters, GE, Fat mimetics, Inulin, Pectin, Shortbread

## Abstract

Shortbread biscuits have become one of the most desirable snacks in all population groups but due to its high fat content it is searching for new solutions of fat reduction such as the use of carbohydrate-based fat mimetics. However, the thermal processing of food containing lipids in the presence of certain carbohydrates can contribute to the formation of toxic compounds or alter its levels. Therefore, the aim of the study was to evaluate the use of inulin and pectin gels as partial fat replacers (10–40%) in classical shortbread biscuits in a view of the changes of 3-monochloropropane-1,2-diol ester (3-MCPDE) and glycidyl ester (GE) levels after baking, and also after a two-months storage. The experiment showed that the amount of inulin gel higher than 10% promoted the endogenous formation of 3-MCPDE probably due to the higher amount of Maillard products formed in the biscuits, but after two months the levels decreased and were comparable with those transferred from shortening. Pectin did not demonstrate such phenomenon, owing to the presence of bound water in gel. Glycidyl esters were partially decomposed during baking and also during storage, particularly in the samples with the addition of pectin gel, which presumably caused by low stability of GE under acidic conditions resulting from the occurrence of galacturonic acid in pectin.

**Electronic supplementary material:**

The online version of this article (10.1007/s11130-019-00723-z) contains supplementary material, which is available to authorized users.

## Introduction

Shortbread biscuits have become one of the most desirable snacks in all population groups due to their low manufacturing cost, more convenience, and long shelf-life, up to 10 months [1]. Nonetheless, the main problem of such products is their high fat and sugar content, which turns them into high-calorie products. What is more, food producers usually use less expensive shortenings, which are based on palm fat that is rich in saturated fatty acids, mainly palmitic acid [2]. High quantities of saturated lipids have been associated with serious health problems such as obesity, cancer, high blood cholesterol and coronary heart diseases [3–5]. Therefore, the formulation of low-fat products has become a high priority for food industry. In addition, nowadays consumers are much more concerned about their health and demand food of natural origin, conferring also health benefits. Hence, it is searching for new solutions to produce pro-health foods that meet the consumers’ expectations [3, 6, 7].

In shortbread fat plays an important role in improving palatability, texture and flavour and its fully replacement has become a challenge for scientists and food technologists [8]. There are different approaches for saturated fat reduction in bakery items, such as substitution with vegetable oils or the use of fat mimetics (protein- and carbohydrate- based) – gel-like matrices that are formed at the presence of substantial levels of water, resulting in lubricant and flowing properties similar to those of fats [4]. Recently, the use of carbohydrate-based fat mimetics such as inulin or pectin has become a popular concept of fat level reduction in bakery products. Inulin is a heterogeneous blend of fructose polymers derived from chicory root, garlic, wheat, bananas, and artichokes. It has been reported as a potential ingredient to imitate the functional and sensorial properties of fat and sugar, at the same time providing high-quality baked products with considerably fewer calories [8]. Inulin also possesses prebiotic properties, which are linked to a variety of beneficial physiological effects as improving bowel habits, increasing calcium absorption, lowering of serum lipids, a positive effect on feeling of satiety, and stimulating the immune system [9]. Up to now, inulin has been successfully applied as a fat replacer even up to 50% without any substantial changes in sensory characteristics of cookies [8, 10]. Pectins, the methyl esters of polygalacturonic acid and its salts, are complex natural carbohydrates found in plant cell walls, especially in apples and citrus fruits. Due to strong gelling and thickening properties, pectin has been used extensively in a variety of foods in order to control the texture and rheology. Another advantage of pectin is good stability in high temperatures [11, 12]. Its beneficial health effects such as anti-inflammatory, anti-cancer, and hypocholesterolemic activities have also been reported. So far, pectins were evaluated as an effective fat replacer in biscuits up to 30% without a loss of quality [13, 14].

The presence of vegetable fat such as palm oil in food is also related to the risk of the occurrence of some contaminants that can be transferred with fat to food products. It has been shown that refined edible oils might contain high levels of esters of higher fatty acids and 3-chloropropane-1,2-diol (3-MCPD) (3-MCPD esters, 3-MCPDE) or glycidol (glycidyl esters, GE). These compounds, which are similar in their structure to lipids, are formed during refining of vegetable oil [15, 16]. According to EFSA report [17], the ester levels in vegetable fats range from 181 to 2912 μg kg^−1^ (3-MCPDE, expressed as free 3-MCPD) and from 114 μg kg^−1^ to as much as 3955 μg kg^−1^ (GE, expressed as glycidol moiety). Therefore, partially replacement of vegetable fat with other substances in bakery items can be a simple mean for the decreasing MCPDE and GE in human diet. On the other hand, the formation of esters during thermal processing of food has been reported recently [18]. Thus, the occurrence of esters in bakery commodities can origin from bakery fat or the compounds can be generated during heat treatment. However, no such study for bakery items with reduced fat level has been performed in available literature.

For that reason, in the presented paper we decided to evaluate the use of inulin and pectin as partial fat replacers (10–40%) in classical shortbread biscuits in a view of the changes of 3-MCPDE and GE levels after baking, and also after two-months storage.

## Materials and Methods

### Biscuit Ingredients

Inulin (derived from chicory root) was purchased from Intenson, Poland. Apple pectin (non-amidated) was delivered by Naturex Group, Poland. Other biscuit ingredients (wheat flour type 500, bakery fat, eggs, sucrose) were purchased from a local supermarket. The bakery fat (total fat level 80%) contained sunflower and rapeseed oil in varying proportions, palm oil, rapeseed oil partially hydrogenated, water, acidified milk, salt (0.3%), sugar, emulsifiers (mono- and diglycerides of fatty acids, mono- and diglycerides of fatty acids esterified with citric acid, sunflower lecithin), aroma, annatto, citric acid, vitamins A and D_3_ (according to the manufacturer).

### Formulation of Biscuits

The biscuit formulation was based on a standard shortbread recipe (Table 1). For bakery fat substitution inulin (samples marked as “I”) or pectin (“P”) gels were prepared by dispersing inulin and pectin powders in distilled water, at the minimum concentrations that resulted in gel formation: 60% (*w/w*) for inulin and 20% for pectin. The gels were further used as fat replacement at following quantities: 0 (control), 10, 20, 30, and 40%. Therefore, 9 biscuit recipes were prepared, each in four independent replicates. After thorough mixing all ingredients, the dough was cut into 0.5 cm thick discs of 5 cm diameter, weighed, and then baked at 200 °C for 10 min in a Hendi G5D convection steam oven (Hendi Food Service Equipment, Netherlands). After baking, the biscuits were cooled to ambient temperature, weighed once again, and divided into two groups. The first group was analysed immediately after baking, while the second group of biscuits was packed in polyethylene bags, tightly sealed and stored for two months. The storage experiment was conducted in an air-conditioned laboratory under storage conditions according to the standard [19].

**Table 1:** Dough ingredients used in examined variants of the biscuits in the study

Ingredients [g]	Control (K)	Biscuits with inulin				Biscuits with pectin			
		10% (I 10)	20% (I 20)	30% (I 30)	40% (I 40)	10% (P 10)	20% (P 20)	30% (P 30)	40% (P 40)
Wheat flour	300	300	300	300	300	300	300	300	300
Bakery fat	200	180	160	140	120	180	160	140	120
Inulin gel	–	20	40	60	80	–	–	–	–
Pectin gel	–	–	–	–	–	20	40	60	80
Sucrose	100	100	100	100	100	100	100	100	100
Yolk eggs	3	3	3	3	3	3	3	3	3

### Determination of 3-MCPDE and GE

Determination of 3-MCPD esters and glycidyl esters was performed in all samples of biscuits, immediately after baking and after a two-months storage period. All dough ingredients were also analysed for 3-MCPDE and GE levels. The extraction and determination of fat was performed using CO_2_ in critical phase, according to the procedure formerly developed and validated [20]. The extracted fat was spiked with 270 μL of PP-3-MCPD-d_5_ and P-Gly-d_5_, used as internal standards. Further steps were performed according to the procedures described previously [20].

### Determination of Water Content

Water content of the biscuits was determined by the weight-loss method as described in the standard [21]. The samples were dried in an oven at 130 °C until constant weight was achieved.

### Changes of Fat Replacers upon Heat Treatment

200 mg of pectin and inulin gels were heated at 200 °C for 10 min. After cooling, the resulting residues were weighted and 10 mL of deionised water was added; the samples were vortexed for 2 min and filtered with a filter paper. The extracts were further used for the determination of pH level. pH level was also determined for raw inulin and pectin at the concentration of 2% (*w/w*).

## Results and Discussion

### The Changes of the Ester Levels during Baking

**Fig. 1:**
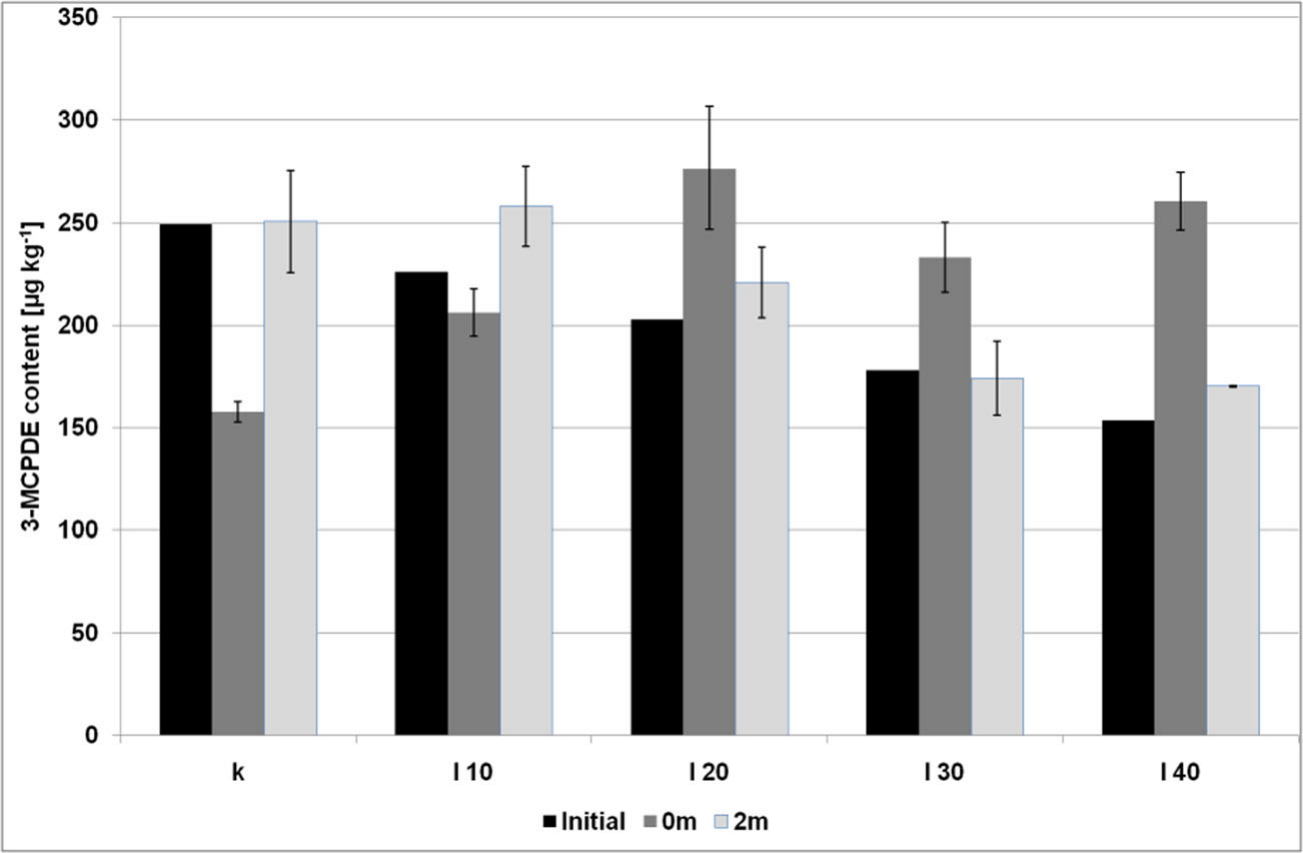
The content of 3-MCPDE (expressed as free 3-MCPD) in the biscuits with the inulin gel addition; initial – the levels of 3-MCPDE transferred from the bakery fat to biscuits (calculated theoretically); 0 m – the levels of 3-MCPDE in the biscuits after baking; 0 m – the levels of 3-MCPDE in the biscuits after a two-months storage; error bar is a confidence interval (95%)

**Fig. 2:**
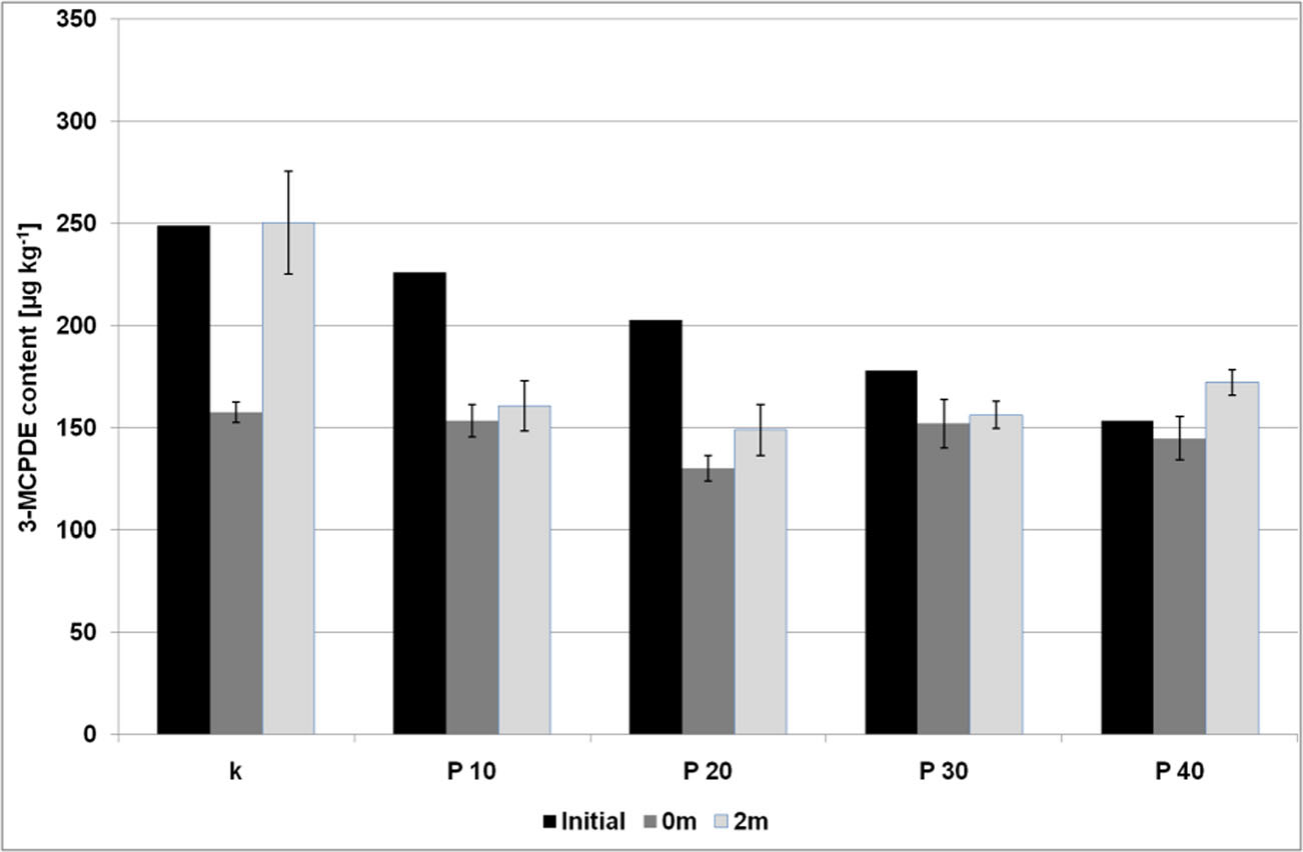
The content of 3-MCPDE (expressed as free 3-MCPD) in the biscuits with the pectin gel addition; initial – the levels of 3-MCPDE transferred from the bakery fat to the biscuits (calculated theoretically); 0 m – the levels of 3-MCPDE in the biscuits after baking; 0 m – the levels of 3-MCPDE in biscuits after a two-months storage; error bar is a confidence interval (95%)

**Fig. 3:**
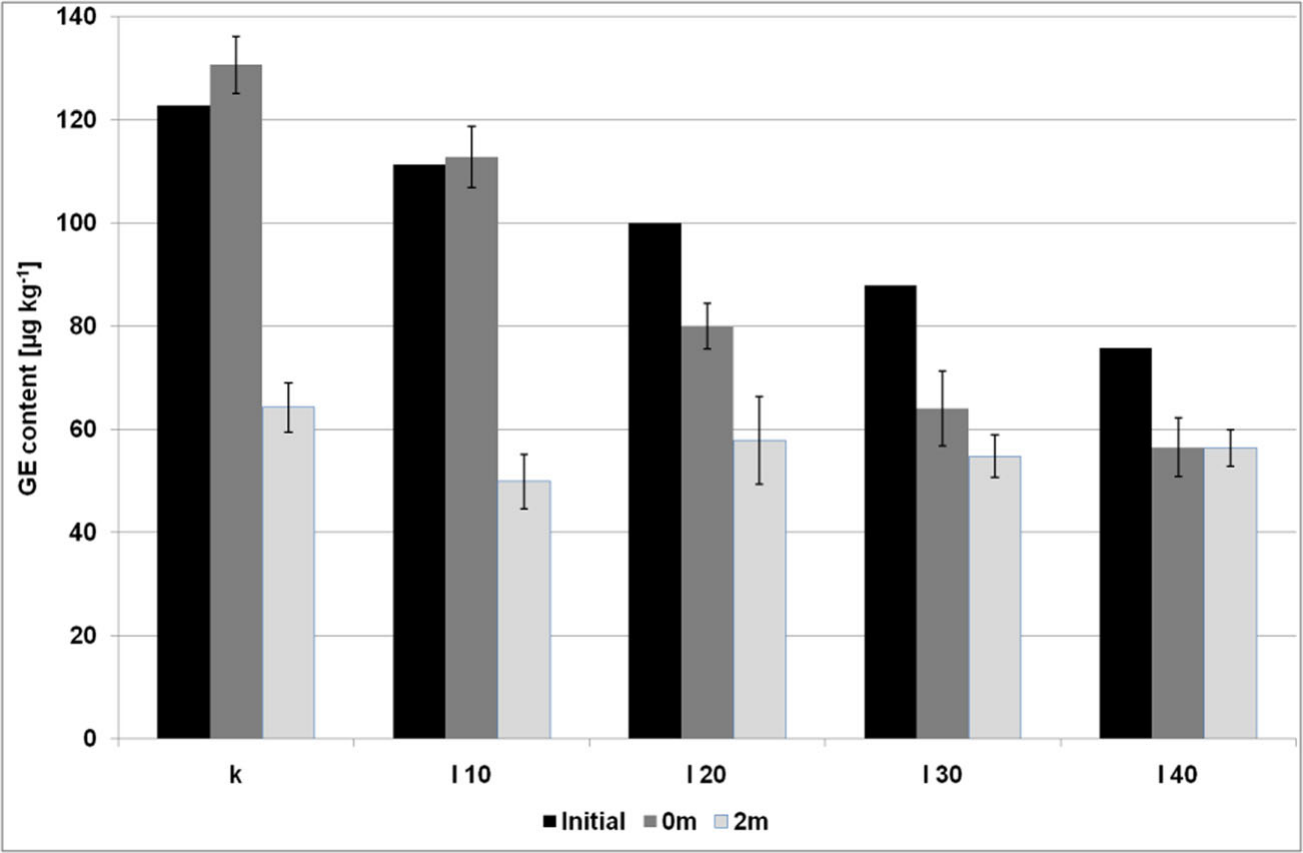
The content of GE (expressed as glycidol moiety) in the biscuits with the inulin gel addition; initial – the levels of GE transferred from the bakery fat to the biscuits (calculated theoretically); 0 m – the levels of GE in the biscuits after baking; 0 m – the levels of GE in the biscuits after a two-months storage; error bar is a confidence interval (95%)

**Fig. 4:**
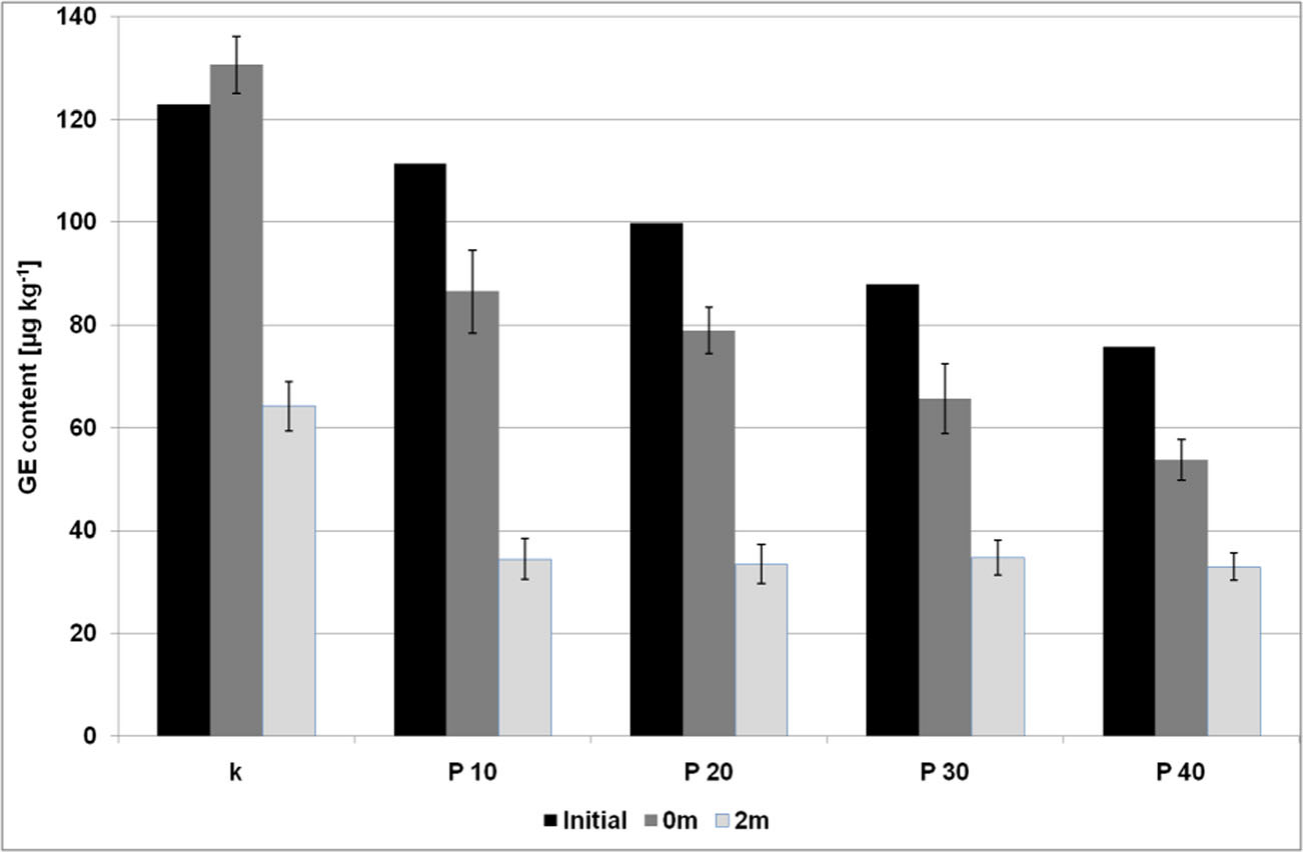
The content of GE (expressed as glycidol moiety) in the biscuits with the pectin gel addition; initial – the levels of GE transferred from the bakery fat to the biscuits (calculated theoretically); 0 m – the levels of GE in the biscuits after baking; 0 m – the levels of GE in the biscuits after a two-months storage; error bar is a confidence interval (95%)

The 3-MCPDE level in the fat phase of bakery fat was equal to 905 ± 15 μg kg^−1^ (mean and standard deviation of four independent replicates), while the glycidyl esters reached 446 ± 21 μg kg^−1^ (expressed as glycidol moiety). Other dough ingredients were free of examined esters. The fat amount in baked biscuits ranged from 17.3 to 26.2% (*w/w*), with the highest level found in the control sample and the lowest one in the samples with the use of 40% of fat replacer, which was in good agreement with the values calculated theoretically (Fig. 1S and Fig. 2S – Supplementary Material). Based on these results, it was established that the baked biscuits (including also the loss of water during baking) should contain from 166 to 263 μg kg^−1^ of 3-MCPDE and 82–130 μg kg^−1^ of GE and the amount of both esters should decrease with the reduction of the shortening content (Figs. 1, 2, 3, and 4). Meanwhile, the highest amount of 3-MCPDE was detected in the sample that contained 20% of inulin gel instead of fat (I20, 277 μg kg^−1^); the lowest level was discovered in the sample with 20% of pectin (P20) and was equal to 130 μg kg^−1^. Glycidyl ester levels ranged from 54 μg kg^−1^ (I40) to 131 μg kg^−1^ (control sample).

The levels of glycidyl esters were significantly lower (*p* < 0.05 in one-way analysis of variance) from the calculated theoretically values in all samples, except for the sample I10. The most significant difference (at about 34%) was observed in the sample P40. No increase in GE amount during baking was observed. GE slightly increased only in the control sample, at about 6%. The amount of GE detected in the samples was well correlated with the decreasing amount of bakery fat (correlation coefficient equal to 0.97 and 0.93 for the samples with inulin and pectin, respectively). This implies that glycidyl esters were partially decayed during baking, and probably no other factors affected this process.

For the 3-MCPD esters its levels differed significantly from the theoretical values in almost all investigated samples, except for the sample P40. In three samples with inulin gel (I20, I30, I40) the 3-MCPDE amount raised, and for the sample I40 this increase was equal to 57%. On the contrary, in the control sample and the samples with pectin gel (P10, P20, P30) the decrease in 3-MCPD content was reported and the most substantial change was observed for the control and P20 (40%). What is more, no correlation between fat amount and 3-MCPDE levels similar to those reported for GE was noted. This indicates that the 3-MCPD esters present in bakery fat were transferred to dough but high temperatures and the presence of inulin and pectin altered its level. Indeed, the inulin gel in the amounts higher than 20% contributed to increased levels of 3-MCPDE, which were even higher than those observed in the control sample. This phenomenon suggests that the presence of inulin can lead to the additional formation of 3-MCPDE.

Among the samples with the use of pectin gel the levels of 3-MCPDE were comparable. No statistically significant differences were observed between the 3-MCPD amounts in the control sample and the samples with the pectin gel. Hence, in the control sample, the 3-MCPD esters were probably partially decayed during baking and in the rest of the samples with pectin the degradation also occurred, but in the latter case the degradation process was slower.

### The Changes of Water, Fat and Ester Levels after Storage

The amount of water as well as fat content did not change significantly during two months of storage (Fig. 1S, 2S and 3S – Supplementary Material), while in the case of 3-MCPD esters statistically significant changes were observed for all samples with the use of inulin (Fig. 1). The amount of 3-MCPDE increased about 59 and 25% for the control sample and the sample I10, respectively, while for the rest of the samples (I20, I30, I40), the contents of 3-MCPDE were lower than before the period of two months, and, surprisingly, were roughly comparable to those calculated from bakery fat. The most significant decrease was discovered for the sample I40 (35%). It appears therefore that the 3-MCPD esters were decayed upon storage probably with the release of free 3-MCPD. In opposite to these findings, among the samples with pectin only the sample P40 exhibited significant change in the level of 3-MCPDE – an increase at about 20%, comparing to the amount after baking (Fig. 2). Glycidyl esters decreased significantly in the control sample, all samples with the addition of pectin and in the samples I10 and I20 (Figs. 3 and 4). GE amount maintained at the same level after storage only in the samples with the level of inulin higher than 20%.

### Influence of Inulin and Pectin on 3-MCPDE and GE Levels

Differentiated levels of 3-MCPDE and GE resulting from the use of two fat mimetics can be explained by its physicochemical properties and its changes upon heat treatment. In order to explain the changes in its properties during thermal processing, inulin and pectin gels were submitted to heating at 200 °C for 10 min and then pH level was measured. pH values of raw inulin (2% solution) was equal to 5.52 ± 0.09 but after heating it decreased to 4.21 ± 0.15 as the effect of thermal degradation of sugars. pH of raw pectin was significantly lower (2.08 ± 0.15), which resulted mainly from the presence of galacturonic acid. After heating pH did not change significantly and was equal to 1.95 ± 0.11.

Differences in loss of mass during heating were another interesting observation. In the case of inulin gel, which contained 60% dried inulin and 40% water, the loss of mass was reported to be about 63%. This value resulted not only from the water evaporation but could also arise from the thermal degradation of inulin. For pectin gel although it contained more water (80%) and less dried pectin (20%), the loss of mass was only about 20%, which means that upon heating water was not completely evaporated and still remained in the gel. Therefore, it can be concluded that pectin gels are more effective in binding water and are more stable to high temperatures, in contrary to inulin. Inulin is composed with long fructose chains, which during thermal treatment are readily decomposed to new products, mainly fructose and di-D-fructose dianhydrides [22, 23]. This process accelerates the non-enzymatic browning of dough leading to the formation of Maillard compounds [24, 25]. Therefore, the biscuits with higher levels of inulin usually show darker colour, which was also observed in this study, especially for the sample with 40% use of inulin. Maillard compounds and organic acids, which are formed from reducing sugars, are also supposed to promote the formation of 3-MCPD in food [26]. Pectin is whereas much more stable during heating and, additionally, it has been observed that hydrocolloids based on pectin are able to maintain water and can also inhibit the formation of Maillard compounds [13, 27]. Therefore, the cakes with the use of pectin as fat replacer usually show lighter colour [13]. This observation was also noticed in this experiment, but only for the sample with 40% use of pectin. Apart from lower Maillard product formation higher water activity in the samples with pectin could probably prevent from the endogenous formation of the 3-MCPDE upon heating.

On the other hand, low pH level of pectin might be a potential explanation for the decay of glycidyl esters during heating and storage. It is well known that GE are unstable due to the presence of an epoxide group in its structure, which can be destructed under acidic conditions [16]. Hence, the acids in the samples with the addition of pectin might accelerate the degradation of glycidyl esters upon storage. Decay of GE over a period of few months has already been reported [28].

## Conclusions

The obtained results reveals that the presence of inulin or pectin added instead of some fat amount can influence on the levels of 3-MCPDE and GE in shortbread during its baking and storage. The amount of inulin higher than 10% can promote the endogenous formation of 3-MCPD esters. However, the esters in the samples with higher amount of inulin are decayed during a long-term storage. Second investigated fat replacer, pectin, does not demonstrate such phenomenon; no endogenous formation of 3-MCPDE has been observed and the levels of 3-MCPDE were more stable during storage. Presented experiment also indicates that the presence of acids can influence on GE amount in shortbread, while water has a substantial impact on the amount of 3-MCPDE.

From consumers’ point of view, the partial replacement of bakery fat with inulin or pectin can be an effective mean to decrease high energy value of shortbread and eliminate the presence of saturated fat from the diet. Both inulin and pectin have many pro-healthy properties which make them an interesting ingredient for many food products. However, regarding thermal degradation of the inulin it should be emphasized that consumption of the thermal processed products containing more than 20% inulin use as fat replacer should not be recommended.

## Electronic supplementary material

ESM 1 (DOC 250 kb)

## Abbreviations

3-MCPD: 3-monochloropropane-1,2-diol
3-MCPDE: 3-monochloropropane-1,2-diol esters
GE: glycidyl esters
