# Supplementary material for: Carbohydrate-Based Fat Mimetics Can Affect the Levels of 3-Monochloropropane-1,2-Diol Esters and Glycidyl Esters in Shortbread Biscuits
Source: Plant Foods Hum Nutr. 2019 Mar 5;74(2):216–22. doi: 10.1007/s11130-019-00723-z (PMC6525143; doi:10.1007/s11130-019-00723-z)
Supplement: Supplementary file 1 — (DOC 250 kb) [file 11130_2019_723_MOESM1_ESM.doc]

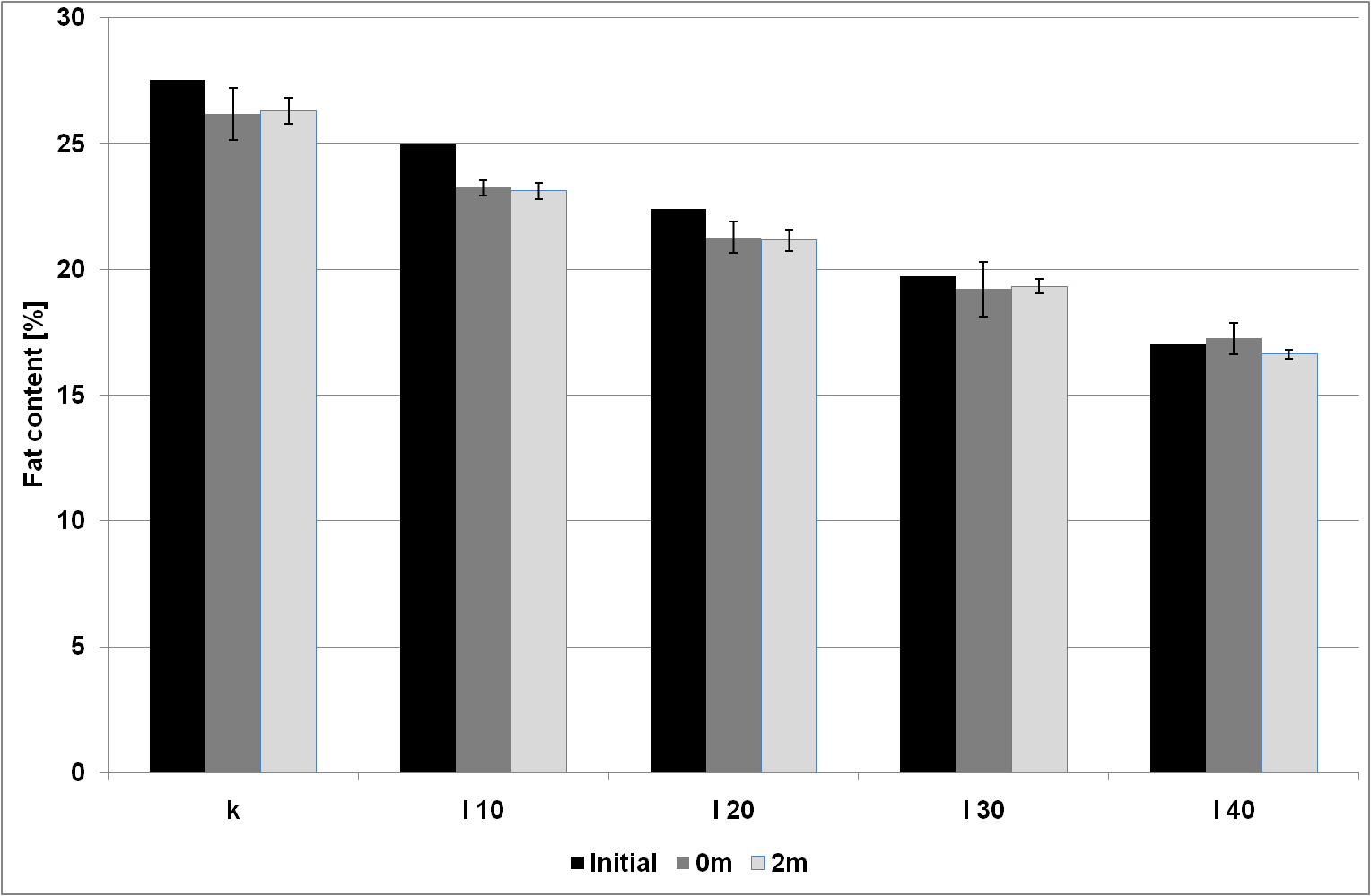


**Fig. 1S** The content of fat in the samples with the use of inulin gel; initial – fat content transferred from the bakery fat to the biscuits (calculated theoretically); 0m – fat content after baking, 2m – fat content after 2-month storage; error bar is a confidence interval (95%)


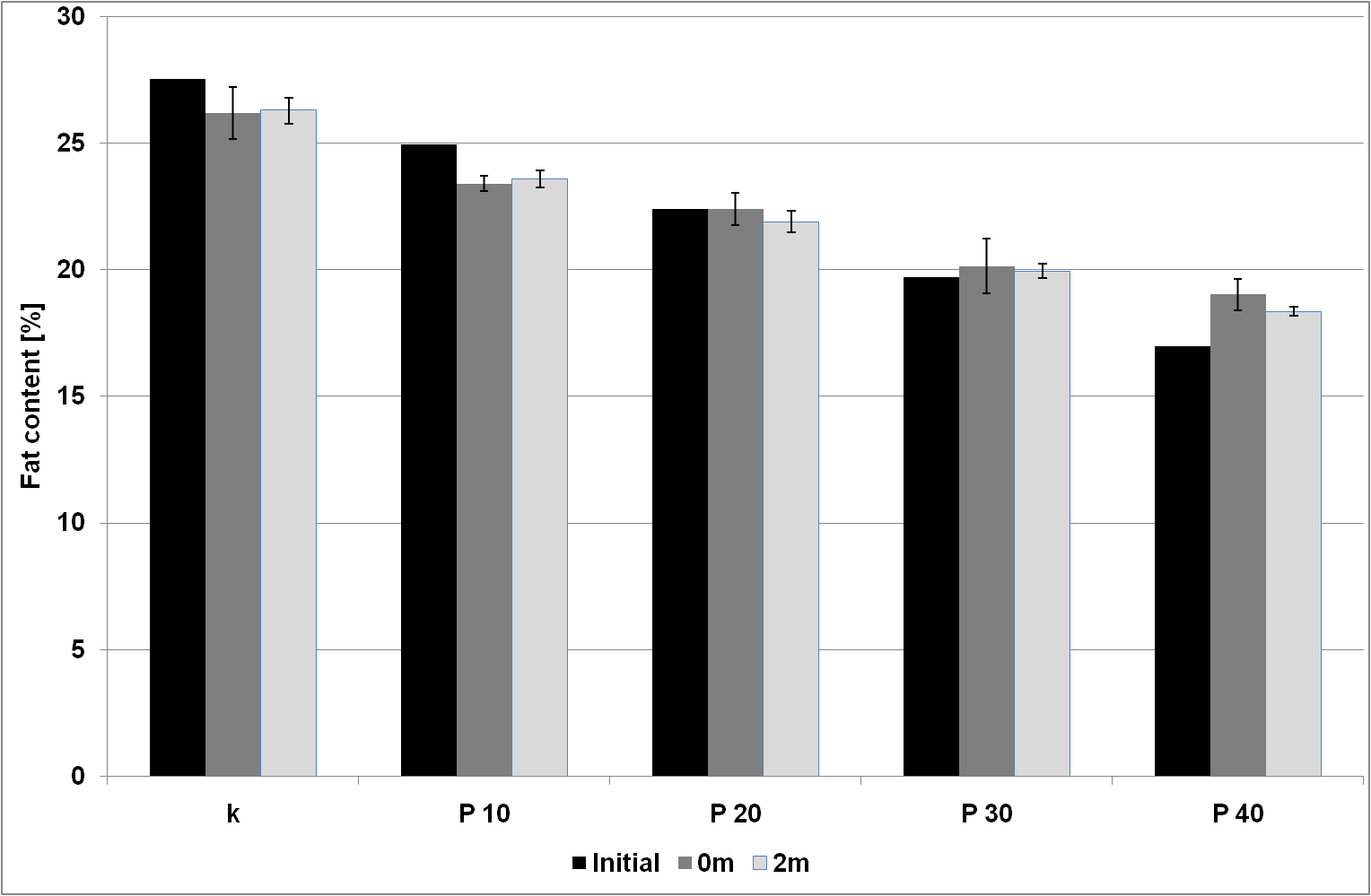


**Fig. 2S** The content of fat in the samples with the use of pectin gel; initial – fat content transferred from the bakery fat to the biscuits (calculated theoretically); 0m – fat content in biscuits after baking, 2m – fat content in biscuits after 2-month storage; error bar is a confidence interval (95%)


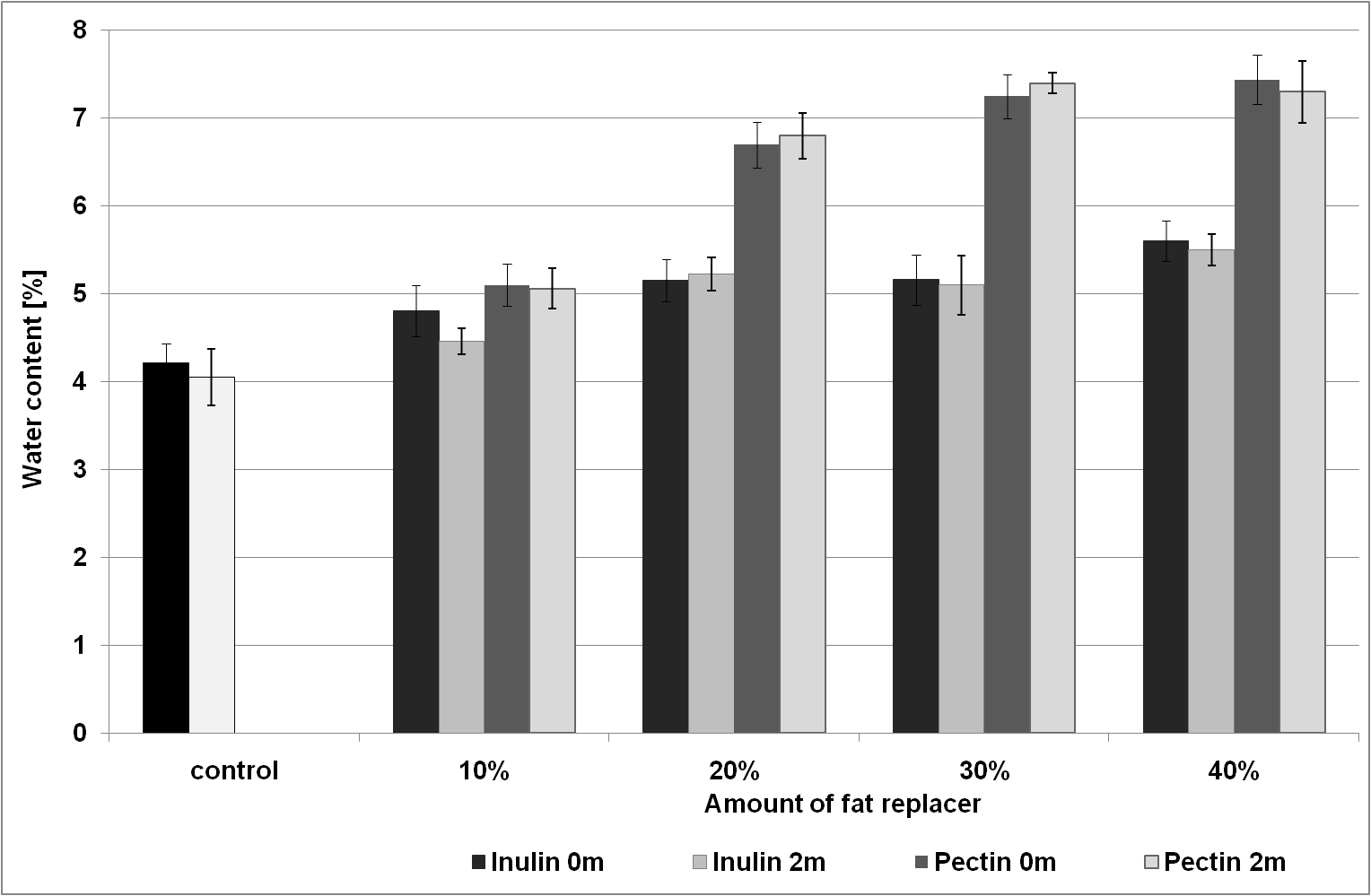


**Fig. 3S** The content of water in the biscuit samples; 0m – after baking, 2m – after 2-month storage; error bar is a confidence interval (95%)
